# A reference model based interface terminology for generic observations in Anatomic Pathology Structured Reports

**DOI:** 10.1186/1746-1596-9-S1-S4

**Published:** 2014-12-19

**Authors:** Gunter Haroske, Thomas Schrader

**Affiliations:** 1 Institute of Pathology, Dresden-Friedrichstadt General Hospital, Dresden, Germany; 2 Department Informatics and Media, University of Applied Sciences, Brandenburg, Germany

## Abstract

**Background:**

Current terminology systems for structured reporting in pathology are more or less focused on tumor pathology. They have not been compiled in a systematic approach, therefore they gather terms of very different granularity. Generic models for terminology development could help in establishing reference terminologies for all fields of anatomic pathology.

The core principle of those models is the ontological structure of native speaking terminology. By analyzing the PathLex interface a generic terminology model will be derived.

**Methods:**

For each element template of PathLex its possible generic nature and its value set was analyzed, looking for the uniqueness or multiplicity of the values in the value sets.

The generic terms were mapped to SNOMED-CT terms using "ArtDecor".

**Results:**

The 488 PathLex element templates for Anatomic Pathology (AP) observations can be reduced to 53 generic templates, leaving out only 17 templates very specific for organ and/or disease. Among those 53 templates 28 are describing UICC-TNM staging, ICD-O-classification, and grading. Further 15 templates describe the results from marker investigations. Almost all of the terms, used in those templates could be mapped to SNOMED CT.

All of the generic elements have their "organ specific" counterparts by assigning them to one of 20 organs and invasive or noninvasive cancer, respectively. Studying the structure of generic and specific terms it becomes obvious that any AP observation

- occurs always in a context

- consists of three basic elements (target of observation, property of observation, additional qualifiers, added by value sets for coded data).

**Conclusions:**

If a machine-readable terminology is aimed to preserve all the information of native speaking, then two principal solutions exist:

- ystematic consideration of all the aspects mentioned above in each single term

- ocusing on the generic elements of terms and combining this with the structure of communication, reflecting the non-obvious elements of the terminology.

The fastest way for establishing an interface terminology is the first approach, which lists all of the terms needed for e.g. a checklist in a comprehensive manner (precoordination).

However, if the list of terms and problems increases, or new requirements have to be met, considerable difficulties may arise in keeping the terminology consistent and complete.

The second, postcoordination approach offers some advantages. It does not have limitations in the organ- or disease specificity, and it keeps the number of terms limited, making them more easily to survey.

## Background

"To benefit from a terminology, it must be implemented and used as part of an application" [1].

As long as reference terminologies such as SNOMED-CT are not widely available for end-users in record services, i.e. functions that involve storing, retrieving or processing application data,

interface terminologies are the best solution to bridge this gap. Current interface terminology systems for structured reporting in pathology, however, are more or less focussed on tumor pathology, and closely oriented on widely used templates for data entry, e.g. CAP cancer checklists. They are part of the model of use and have not been compiled in a systematic approach, therefore they gather terms of very different granularity and focus of interest. As to enable a systematic approach for establishing rules for terminology development reference terminology models may help. This should a mapping to reference terminologies as part of the model of meaning make easier [2,3].

The core principle of those models is the ontological structure of both "native speaking" terminology in anatomic pathology and its scientific background in the ontology of systemic pathology. The question is what are the generic aspects of each observation in anatomic pathology?

By analyzing the PathLex interface terminology [4] and comparing it with the SNOMED-CT reference terminology, such a generic reference terminology model will be derived. It describes a framework of classes of terms and the relationships between them as to represent concepts. This reference model then can serve as a basis for a style guide for defining new terms in all fields of anatomic pathology.

## Methods

The Pathlex interface terminology is accessible so far by an EXCEL sheet [5]. A special software tool "termAPP" makes its internal relationships more visible [6]. For each element template its possible generic nature and its value set was analyzed, looking for the uniqueness or multiplicity of the values in the value sets.

The generic terms were mapped to preexisting SNOMED-CT terms. For SNOMED-CT a broad spectrum of evaluation and application tools exist. "Art-Decor" [7] was used for this study.

## Results and discussion

Due both to the complexity of SNOMED CT and gaps in LOINC and SNOMED CT for their use in recent anatomic pathology daily practice an interface terminology PathLex was developed by the IHE Anatomic Pathology Working Group [8]. As interface terminologies in general, also PathLex is primarily aimed to enable an easy data entry at the front end of Pathology Reporting Systems. However, with increasing coverage of fields of anatomic pathology, such an interface terminology needs more rules and reference models than PathLex recently is based on.

The 488 PathLex element templates for Anatomic Pathology (AP) observations describe so-called "clinical statements", representing

- 467 observations, which may refer to specimens, lesions, morphological items, results of special techniques, e.g. immunohistochemistry or molecular pathology, results of measurements, disorders, prognosis, staging and grading, and classification

- 21 procedures, which may refer to specimens and images,

The 467 observation terms can be condensed to 53 generic terms, leaving out only 17 terms very specific for organ and/or disease. Among those 53 terms 28 are describing UICC-TNM staging, ICD-O-classification, and grading. Further 15 terms describe the results from marker investigations. For those groups of terms reference models are already used [9]. There are only 9 terms with a really generic scope for an AP observation. PathLex terms with their generic equivalents are shown in table 1.

**Table 1:** Equivalent generic terminology elements in PathLex.

APSR template id	APSR element name (PathLex)	Generic element
e.g. 1.3.6.1.4.1.19376.1.8.1.4.54	e.g. Cytological type	Class
e.g. 1.3.6.1.4.1.19376.1.8.1.4.164	e.g. Lesion size, largest dimension	Diameter
1.3.6.1.4.1.19376.1.8.1.4.146	Distance of lesion from closest uninvolved margin	Distance
1.3.6.1.4.1.19376.1.8.1.4.162	Treatment effect	Treatment Effect
1.3.6.1.4.1.19376.1.8.1.4.149	Extent	Extent
1.3.6.1.4.1.19376.1.8.1.4.143	Lesion focality	Focality
e.g. 1.3.6.1.4.1.19376.1.8.1.4.151	e.g. Histological grade (WHO)	Grade
1.3.6.1.4.1.19376.1.8.1.4.168	Macroscopic type	Growth pattern
1.3.6.1.4.1.19376.1.8.1.4.174	Specimen integrity	Integrity
e.g. 1.3.6.1.4.1.19376.1.8.1.4.141	e.g. Margins involvement	Involvement
e.g. 1.3.6.1.4.1.19376.1.8.1.4.439	e.g. Estrogen receptor	Marker
e.g. 1.3.6.1.4.1.19376.1.8.1.4.318	e.g. Lesion ulceration	Neighborhood relationship
e.g. 1.3.6.1.4.1.19376.1.8.1.4.156	e.g. Number of lymph nodes involved	Number
e.g. 1.3.6.1.4.1.19376.1.8.1.4.148	e.g. Lymph node sampling	Procedure
e.g. 1.3.6.1.4.1.19376.1.8.1.4.169	e.g. Lesion site	Site
e.g. 1.3.6.1.4.1.19376.1.8.1.4.161	e.g. pT	Stage
1.3.6.1.4.1.19376.1.8.1.4.160	Specimen weight	Weight

Most of the generic elements in Pathlex have their "organspecific" counterparts by assigning them to one of 20 organs and, additionally, to invasive or noninvasive cancer, respectively. In this way the context information (organ, invasive or noninvasive cancer) became part of the terminology itself. The analysis shows also that PathLex templates do not reflect relationships to each other except their organ and disease specificity.

About two third of those generic PathLex terms could be mapped to SNOMED CT. Recently there is a coverage of 60,3%.

By this mapping it became obvious that there had been different approaches in developing PathLex and SNOMED-CT. Whereas SNOMED-CT is based on concepts, PathLex so far is rather a compilation of terms with their accompanying value sets occurring in the daily routine of pathology reporting or demanded by cancer check lists. From the terminology view point the sets for coded values should be regarded as terms, too. They have not been analyzed in this study.

Studying the structure of generic and specific terms it becomes clear that any AP observation

- occurs always in a context of location, pathological problem and observation method,

- consists of three basic elements (target of observation, property of observation, additional qualifiers and permissible values, which again may be generic terms).

Taking the ontological background of native speaking terminology in anatomic pathology as a starting point, a generic reference terminology model should therefore consist of those four basic elements mentioned above, added by a locator as well as a problem organizer element, and the appropriate relationships. It is directed to an approach known as post-coordination in SNOMED-CT.

**Figure 1:**
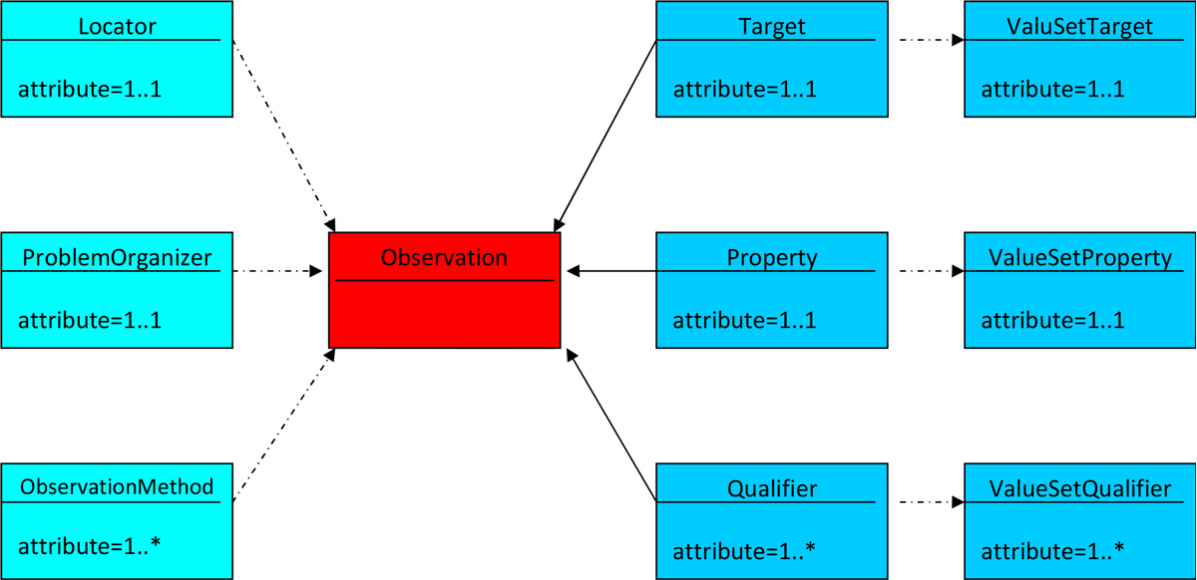
**Terminology reference model for generic AP Observation**. Left: Context Right: Basic elements

**Figure 2:**
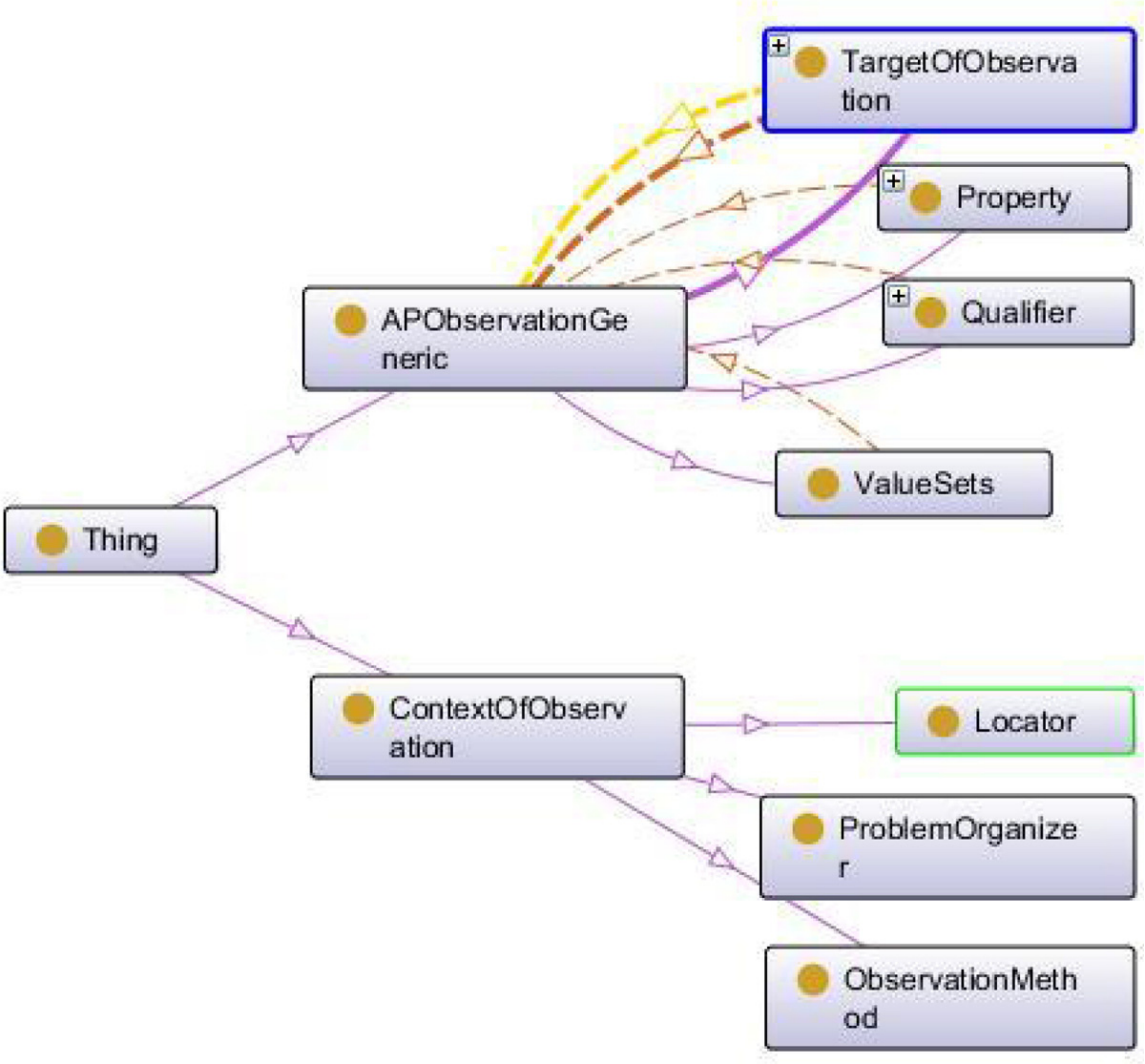
**Ontology for the reference model of generic AP Observation (Protégé)**. Relationships are colour-coded: - pink: IS A - brown: IS PART OF - yellow: IS TARGET OF

**Figure 3:**
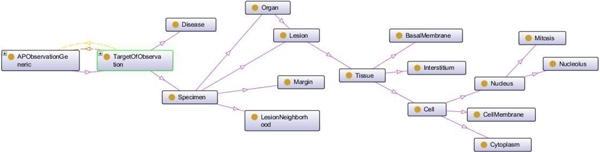
**Ontology of Targets of Observation (Protégé)**.

In Figure 1 a very simple sketch of this reference terminology model is given. In Figures 2 and 3 the ontological relations in and between the basic elements of the reference model are illustrated. In tables 2 through 4 the targets, properties, and qualifiers are shown, which result from the generic PathLex elements added by further basic expressions of general pathology. Their permissible values (concepts and concept descendants) as well as mapped SNOMED-CT codes are given in these tables, too. All the "generic" PathLex terms can be expressed by the generic approach completely, as it is the case, too, for the 17 very organ- or disease specific terms.

**Table 2:** Permissible values (their descendants included) of targets for generic AP observations, might be extended.

ObservationTargetName *(SNOMED-CT Axis)*	SNOMED-CT code
*Cell*	*362837007* *(cell structure)*
*Disease*	*64572001* *(disorder)*
*Lesion*	*49755003* *(morphologic abnormality)*
*Margin*	*82868003* *(body structure)*
*Mitotic nucleus*	*49307000* *(cell structure)*
*Nucleolus*	*15982001* *(cell structure)*
*Nucleus*	*84640000* *(cell structure)*
*Organ*	*272625005* *(body structure)*
*Specimen*	*123038009* *(specimen)*
*Tissue*	*85756007* *(body structure)*

SNOMED-CT axis in brackets.

**Table 3:** Permissible values (their descendants included) of properties for generic AP observations, might be extended.

ObservationPropertyName *(SNOMED-CT Axis)Area*	SNOMED-CT code
*Area*	*42798000* *(qualifier value)*
*Class*	*277046005* *(attribute)*
*Colour*	*263714004* *(qualifier value)*
*Consistency*	*246191002* *(attribute)*
*CytologicAtypia*	*50673007* *(morphologic abnormality)*
*Diameter*	*81827009* *(qualifier value)*
*Distance*	*246132006* *(qualifier value)*
*General clinical stage for disease AND/OR neoplasm*	*106240007* *(qualifier value)*
*Grade*	*103421006* *(attribute)*
*Involvement*	*278112009* *(attribute)*
*MarkerStatus*	*246110002* *(attribute)*
*Measurement*	*122869004* *(procedure)*
*NeighborhoodRelationship*	*(408739003)* *(attribute)*
*Number*	*410680006* *(attribute)*
*Pattern*	*255711007* *(attribute)*
*Regularity*	*246202005* *(attribute)*
*ResidualDisease*	*65320000* *(qualifier value)*
*Shape*	*300842002* *(attribute)*
*Size*	*246115007* *(attribute)*
*SpecificDeposits*	*46595003* *(morphologic abnormality)*
*(Treatment)Effect*	*(253861007)* *(qualifier value)*
*Weight*	*272102008* *(qualifier value)*

SNOMED-CT axis in brackets.

**Table 4:** Permissible values (their descendants included) of qualifiers for generic AP observations, might be extended.

ObservationQualifierName	***SNOMED-CT code***
*Laterality*	*272741003* *(attribute)*
*Focality*	
*Invasiveness*	*10179008* *(qualifier value)*
*ScaleType*	*370132008* *(attribute)*
*normalStructure*	*361083003* *(body structure)*
*abnormalStructure*	*49755003* *(morphologic abnormality)*
*specialAttributes, like nearest, biggest, most, least, smallest, etc.*	

SNOMED-CT axis in brackets.

Additional to IS-A- and PART-OF-relationships for the targets and properties following relationships should be used: HAS-TARGET for properties and IS-QUALIFIER-OF for qualifiers. Those relationships can easily be expressed within HL7 templates by XML, too.

The Pathlex term "Breast-In situ neoplasm-Lesion size, largest dimension" (OID 1.3.6.1.4.1.49376.1.8.1.4.442) could be expressed by using that generic reference model as "Diameter" (property) of a "Lesion" (target), qualified as "ObservedByMicroscopicInvestigation"(qualifier) and qualified as "Largest" (qualifier) with locator for "breast" and problem organizer for "non-invasive tumor".

Reference model based terminology approaches have been shown to lead to comprehensive description tools for staging and grading of malignant tumors as well as for assessment and scoring systems [9,10].

Some of the terminology aspects may be hidden in the context of the observation, which always accompanies a communication in native language, too.

If a machine-readable terminology is aimed to preserve all the information of native speaking, then two principal solutions exist:

- systematic consideration of all the aspects mentioned above in each single term (pre-coordination approach)

- focusing of the generic elements of terms and combining this with the structure of communication, reflecting the non-obvious elements of the terminology (post-coordination approach).

The fastest way for establishing an interface terminology is the first, systematic way, which lists all of the terms needed for a data entry device, e.g. a checklist, in a comprehensive manner. In SNOMED-CT this is known as precoordination.

However, if the list of terms and problems increases, or new requirements have to be met, considerable difficulties may arise in keeping the terminology consistent and complete.

Therefore the second, postcoordination approach offers some advantages. It does not have limitations in the organ- or disease specificity, and it keeps the number of terms limited, making them more easily to survey.

Both approaches should be based on an onomasiological approach (concept based approach). With that approach the starting point is always the concept, revealing the existence of synonyms. A concept system allows us to place a previously unknown concept in a semantic context. The core of a concept system, and therefore of the concept based terminology, too, is the system of relationships between the concepts, with generic (IS-A-relationship) as well as partitive (PART-OF-relationships) ones. For practical use "combined" concept systems with generic and partitive relationships are probably the most attractive ones [2].

The development of a terminology will be strongly supported by concept dictionaries and ontologies.

In a representation of such a concept system the generic concepts are always at the top levels whereas the more specific ones are to find further down.

Regarding SNOMED-CT as one of the most comprehensive reference terminologies increasingly used in many countries also an interface terminology should adopt as much of their principles as possible. For anatomic pathology terminology problems are mostly bound to observations and procedures. Observations are found in two top level concepts "clinical finding" and "observable entities", procedures are a top level concept on its own. Clinical findings have been defined as observations, judgements or assessments about patients [11].

Observations in anatomic pathology are done on observable entities. Concepts in this hierarchy can be thought of as representing a question or procedure which can produce an answer or a result [11].

Among the presently 27 children of that top level concept, four of them have special importance for anatomic pathology ("Interpretation of Findings", "Molecular, Genetic, and/or Cellular Observables", "Sample Observables", and "Tumour Observables") with a total of further 149 child concepts.

An observable entity with a result or an answer, being a concept again, is finally a clinical finding. For instance, "Tubule formation score (observable entity)" could be interpreted as the question, "*What is the score for the degree of tubule formation?" *If the score is known, the question is specifically for breast cancer answered by "Majority of tumour >75% (score 1)(finding)".

There are clearly defined rules for the use of attributes for observable entities, clinical findings, and procedures in SNOMED-CT [11]. Those should be regarded for the fine tuning of interface terminologies, too.

In our generic approach SNOMED-CT principles for observable entities are already met. For instance: "Colour of Specimen (observable entity)" in our approach "Colour" is the property of the target "Specimen". However, those rules are not yet fully applicable to anatomic pathology. Therefore it is to recommend deriving the permissible values for targets, properties, and qualifiers as close as possible to existent SNOMED-CT solutions. Sets of rather simple values (e.g. "small", "medium", "large" or "mild", "moderate", "strong") have still to be defined for all of the targets, properties and qualifiers being completed by coded data.

## Conclusion

The usage of generic terms based on reference models for observations reduces the efforts of implementation of them as well as the terminology management. Besides existing reference models for staging and grading of tumors, for assessment scores and interpretation of assessments, a reference model for a generic interface terminology for all fields of anatomic pathology is required.

The generic approach concerns not only the terminology itself, but also the consideration of context information. By the specification of AP observation templates the links to organ and problem should be introduced. The decisive question is, whether a term is generic or specific. The criterion for that is its value set. Having a unique value set in each possible application, the term is generic. If not, than a specific term with its own value set have to be used. If ever possible, generic terms should be preferred. Solutions for procedures, which are closely linked to observations in anatomic pathology, should be worked out soon. The generic approach should help further developing PathLex towards an interface terminology for all fields of anatomic pathology.

## Competing interests

The authors declare that they have no competing interests.

## Notes

Publication of this supplement has been funded by 12th European Congress on Digital Pathology. This article has been published as part of *Diagnostic Pathology *Volume 9 Supplement 1, 2014: Selected articles from the 12th European Congress on Digital Pathology. The full contents of the supplement are available online at http://www.diagnosticpathology.org/supplements/9/S1

## References

[B1] SNOMED-CT Starter Guide 2014 http://www.snomed.org/starterguide_gb.pdf February

[B2] Benson T Principles of Health Interoperability HL7 and SNOMED 2012 Springer

[B3] Rosenbloom ST Brown SH Froehling D Bauer BA Wahner-Roedler DL Gregg WM Elkin PL SNOMED CT to represent two interface terminologies J Am Med Inform Assoc 2009 16 1 81 8 18952944 10.1197/jamia.M2694 PMC2605600

[B4] Daniel C Booker D Beckwith B Della Mea V García-Rojo M Havener L Kennedy M Klossa J Laurinavičius A Macary F Punys V Scharber W Schrader T Standards and specifications in pathology: image management, report management and terminology Stud Health Technol Inform 2012 179 105 22 22925792

[B5] Daniel C Macary F IHE Anatomic Pathology Technical Framework Supplement: Anatomic Pathology Structured Reports (APSR) Trial Implementation Supplement Appendix Value Sets for APSR http://www.ihe.net/Technical_Framework/upload/IHE_PAT_Suppl_APSR_Appendix_Value_Sets_2011_03_31.xls March 31, 2011

[B6] Ouagne D http://termapp.davidouagne.com

[B7] Boers G Heitmann K http://art-decor.org/art-decor/snomed-ct

[B8] Daniel C Macary F Rojo MG Klossa J Laurinavičius A Beckwith BA Della Mea V Recent advances in standards for Collaborative Digital Anatomic Pathology Diag Pathol 2011 6 Suppl 1 17 10.1186/1746-1596-6-S1-S17 PMC3073210 21489187

[B9] Oemig F Lang S Übermittlung onkologischer Daten auf der Basis von CDA R2. HL7-Benutzergruppe in Deutschland e. V 2013 http://wiki.hl7.de/index.php/IG:%C3%9Cbermittlung_onkologischer_Daten

[B10] Altmann U Katz FR Dudeck J A reference model for clinical tumour documentation Studies in Health Technology and Informatics 2006 124 139 144 17108517

[B11] SNOMED-CT Editorial guide 2014 http://www.ihtsdo.org/fileadmin/user_upload/doc/en_gb/eg.html January

